# A prospective multicenter observational study assessing incidence and risk factors for acute blood transfusion reactions in dogs

**DOI:** 10.1111/jvim.17175

**Published:** 2024-09-06

**Authors:** Georgina B. F. Hall, Rachael Birkbeck, Benjamin M. Brainard, Fernanda Camacho, Elizabeth B. Davidow, Dana N. LeVine, Andrew Mackin, Taylor Moss, Katherine J. Nash, Giacomo Stanzani, Daria Starybrat, David Q. Stoye, Carolyn Tai, John Thomason, Julie M. Walker, K. Jane Wardrop, Helen Wilson, Virginie A. Wurlod, Karen Humm

**Affiliations:** ^1^ Clinical Science and Services The Royal Veterinary College London UK; ^2^ The Ralph Marlow UK; ^3^ Department of Small Animal Medicine and Surgery, College of Veterinary Medicine University of Georgia Athens Georgia USA; ^4^ Willows Veterinary Centre and Referral Service Solihull UK; ^5^ Timberline Veterinary Emergency and Specialty Seattle Washington USA; ^6^ Department of Clinical Sciences Auburn University College of Veterinary Medicine Auburn Alabama USA; ^7^ College of Veterinary Medicine Mississippi State University Starkville Mississippi USA; ^8^ School of Veterinary Science University of Queensland Gatton Queensland Australia; ^9^ Dick White Referrals Newmarket UK; ^10^ The Royal (Dick) School of Veterinary Studies University of Edinburgh Edinburgh UK; ^11^ Oxford School of Public Health Oxford UK; ^12^ Cummings School of Veterinary Medicine Tufts University North Grafton Massachusetts USA; ^13^ Department of Medical Sciences University of Wisconsin‐Madison School of Veterinary Medicine Madison Wisconsin USA; ^14^ College of Veterinary Medicine Washington State University Pullman Washington USA; ^15^ Langford Vets University of Bristol Bristol UK; ^16^ Louisiana State University School of Veterinary Medicine Baton Rouge Louisiana USA

**Keywords:** AHTR, dog, FNHTR, leukoreduction, storage lesion, TACO, TRALI

## Abstract

**Background:**

Reported incidence of blood transfusion reactions (TR) varies greatly.

**Objective:**

To prospectively evaluate the incidence of acute TRs in dogs receiving allogenic blood products, using consensus definitions, and to assess factors associated with TRs.

**Animals:**

Dogs (n = 858) administered allogenic blood products (n = 1542) between March and November 2022.

**Methods:**

Prospective, multicenter surveillance study occurring in referral hospitals in the United States, United Kingdom, and Australia recording TRs in dogs administered blood products as defined by the consensus guidelines published by The Association of Veterinary Hematology and Transfusion Medicine in 2021.

**Results:**

The incidence of acute TR was 8.9% (95% CI 7.0‐11.1) for packed red blood cells (pRBCs) and 4.5% (95% CI 2.9‐6.6) for plasma products. The most frequently reported TRs were febrile nonhemolytic TRs (FNHTR; 4%, 95% CI 2.8‐5.5) when administering pRBCs and allergic TRs (3.2%, 95% CI 1.80‐5.10) when administering plasma products. A higher dose of pRBC (adjusted odds ratio [aOR] 1.04 [95% CI 1.00‐1.08]) was associated with a higher odds of TR. Administration of pRBCs stored for longer than 28 days was associated with higher odds of FNHTR (aOR 4.10 [95% CI 1.58‐10.65]) and acute hemolytic TR (AHTR; OR 15.2 [95% CI 3.35‐68.70]) when compared with pRBCs stored for 14 days or fewer. Leukoreduction of pRBC was not associated with lower odds of developing a TR (OR 1.47 [95% CI 0.89‐2.42]).

**Conclusions and Clinical Importance:**

Clinicians should be mindful of the age and dose of pRBC prescribed to dogs.

**Abbreviations**
AHTR: acute hemolytic transfusion reaction
aOR: adjusted odds ratio
AVHTM: Association of Veterinary Hematology and Transfusion Medicine
FFP: fresh‐frozen plasma
FNHTR: febrile nonhemolytic transfusion reaction
LR: leukoreduced
nLR: nonleukoreduced
OR: odds ratio
pRBC: packed red blood cells
RBC: red blood cell
SFP: stored frozen plasma
TACO: transfusion‐associated circulatory overload
TAD: transfusion‐associated dyspnea
TP: thrombocytopenia
TR: transfusion reaction
TRACS: Transfusion Reaction Small Animal Consensus Statement
TRALI: transfusion‐related acute lung injury
WB: whole blood

## 1 INTRODUCTION

Blood product transfusions are vital components for the care of critically ill dogs, but are not without risk. Transfusion reactions (TRs) might cause harm and vary in etiology, severity and time of onset.^1^, ^2^ Literature examining blood transfusions in dogs describes widely varying incidences of acute TRs, ranging between 3.3% and 37.4%, depending on the definition used, blood product administered, study cohort and design.^2^, ^3^, ^4^, ^5^ Reporting of TR incidence and comparing results among studies has been hampered by the lack of consensus definitions for TRs.^2^ In 2021, the Association of Veterinary Hematology and Transfusion Medicine (AVHTM) published a Transfusion Reaction Small Animal Consensus Statement (TRACS). This publication proposed clinical definitions for TRs with the addition of imputability criteria (definite, probable, and possible reaction). Transfusion Reaction Small Animal Consensus Statement further identified current knowledge gaps in the literature and established evidence‐based consensus guidelines for preventing, monitoring, diagnosing, and treating TRs.^2^, ^6^, ^7^

Transfusion medicine practices highlighted by the AVHTM TRACS warranting investigation into their association with TRs in veterinary medicine included prestorage leukoreduction (LR) of red blood cell (RBC) products, packed RBC (pRBC) storage duration, pretransfusion cross‐matching, and the use of premedication.^7^

The primary aim of our study was to prospectively evaluate the incidence of specific acute TRs in dogs receiving allogenic blood products, using TRACS definitions.^2^ The secondary aims were to evaluate the association of the age and dose of RBC units, LR, use of premedication, use of major cross‐matching and dog erythrocyte antigen (DEA)‐1 type‐matching with the incidence of TRs, to identify other transfusion‐related complications, and to document indications for transfusion, transfusion practice and death 24 hours after transfusion for canine allogenic blood products in referral practice.

## 2 MATERIALS AND METHODS

### 2.1 Cases and data collection

Fourteen referral hospitals, 11 academic and 3 private, located in the United States, United Kingdom, and Australia, prospectively logged all allogenic blood products administered to dogs over 9 months (March–November 2022). Transfusions were identified through blood product storage logs, charges on practice management systems, and manual checking of individual units' data. Individual transfusions were recorded in an online questionnaire (Data S1). Briefly, this recorded dog weight, premedication (defined as a pharmaceutical agent administered before the transfusion with the explicit intention of reducing a TR) given, indication for transfusion, survival at 24 hours posttransfusion, collection date and LR status of products, transfusion details including duration, volume administered, transfusion pauses, administration method, pretransfusion cross‐matching, whether the product was DEA‐1 type‐matched, and whether the dog was under general anesthesia (GA) at any point during the transfusion. Possible TRs were logged by recording the presence of any of the following (based on TRACS descriptions of an acute TR): acute fever over 39.2°C (102.5°F) and 1°C (1.8°F) above the temperature at the transfusion start; acute respiratory distress; acute tachycardia, hypotension, or both (systolic blood pressure of <100 mm Hg, with a drop of at least 30 mm Hg from baseline); angioedema, urticaria, pruritus; change in mentation; hypothermia; acute and new‐onset vomiting; acute or new‐onset diarrhea; or ionized hypocalcemia. Any other findings the clinician suspected were consistent with a possible TR were recorded.^2^, ^6^ For any possible reaction, follow‐up questions were asked following algorithms laid out by TRACS, including checking for hemolysis, cardiovascular deterioration, or evidence of cardiac overload.^6^ Treatments or changes to the transfusion plan following a TR were documented.

A transfusion event was defined as the administration of 1 blood product from 1 donor in the case of RBC or plasma products, or a single unit for albumin and platelet products. Blood products were categorized as pRBC, whole blood (WB), plasma products (subcategorized into fresh‐frozen plasma [FFP], stored frozen plasma [SFP], cryoprecipitate, cryo‐supernatant and hyperimmune plasma [Caniplas hyperimmune canine plasma to *Escherichia coli* J5]), platelet products (subcategorized into leukoreduced frozen platelet concentrate, refrigerated platelet concentrate, platelet rich plasma and lyophilized canine platelets), and lyophilized canine albumin.

Indications for using RBC‐containing products were grouped into the following categories: blood loss, hemolysis, ineffective erythropoiesis, extracorporeal treatment, more than 1 indication, other, or unknown. Indications for the use of plasma products were grouped into the following categories: colloidal and volume support, coagulopathy, noncoagulopathy‐induced hemorrhage, extracorporeal treatments, more than 1 indication, other or unknown. Indications for platelet products were grouped into the following categories: immune thrombocytopenia, thrombocytopenia secondary to another etiology, hemorrhage other than due to thrombocytopenia, and extracorporeal treatments.

### 2.2 TR classification

Any potential acute TRs recorded at the time of data entry were examined by 2 authors (GBFH, KH). Transfusion reactions were grouped into the following categories and subcategories: febrile nonhemolytic TR (FNHTR), respiratory reactions (transfusion‐associated dyspnea [TAD], transfusion‐associated circulatory overload [TACO], transfusion‐related acute lung injury [TRALI]), acute hemolytic TR (AHTR), allergic TR, ionized hypocalcemia, and hypotensive TR. Each possible TR was examined and classified according to the case definitions and time frames set out by TRACS.^2^ Imputability (definite, probable, and possible) was assessed by the same authors based on the individual TR definitions.^2^

Broadly speaking, “definite” cases fulfilled specific criteria for the individual TR, with no other cause for the clinical signs identified, “probable” cases either lacked specific data for a definitive classification or the transfusion was thought the most likely cause of the clinical signs, and “possible” cases were when other causes were considered more likely to be the reason for the findings but a TR could not be excluded entirely. Further information was requested if required. If 2 or more transfusions were given on the same day as a TR, the product received at the time of, or closest to, the TR was recorded as the unit associated with the TR. If 2 TR classes could explain the clinical signs seen in 1 transfusion, the records were analyzed, and the TR with the highest imputability was used in the analysis.

Only TRs categorized as definite or probable were included in the analysis to reduce the likelihood of falsely categorizing clinical signs caused by another etiology as a TR.

### 2.3 Statistical analysis

Data analysis was performed using commercially available statistics software (SPSS version 29, IBM), and figures were produced through GraphPad Prism Version 9. Categorical data were presented numerically (percentage), and continuous data were presented as mean (SD) if normally distributed and median (25‐75 percentiles) if skewed. Where clinically pertinent, ranges were reported. For variables with missing data, the size of the denominator population was reported. Incidences of TRs observed within the study sample were presented as percentages. Additionally, 95% Poisson confidence intervals were calculated as estimates of population‐level TR incidence.

Logistic regression was used to test the associations of LR, blood product age, blood product dose, DEA‐1 type‐matched units, pretransfusion cross‐matching, prior transfusion status, administration method, underlying cause of anemia and a transfusion being given under GA, with any TR, FNHTR, AHTR, allergic TR and death. Data were presented as odds ratios (ORs) (95% CI). The effect of the age of pRBC units was assessed with age as a continuous variable and by grouping the units into 0 to 14 days, 14 to 21 days, 21 to 28 days, and 28 to 44 days. Factors that had a *P* < .1 in univariable analysis were then included in a multiple logistic regression to assess for confounding factors with subsequent adjusted ORs (aOR) presented. For anemia type, hemolytic cases were compared with those with blood loss. A sensitivity analysis was conducted excluding the results of 3 dogs who had >1 TR with pRBC, to assess if the inclusion of these animals influenced measures of association between particular exposures and TR types. A further sensitivity analysis was conducted with center added to models to check for confounding by center. Where logistic regression was not possible because of zero TRs occurring in a comparator group, a Fisher's exact test was performed to assess for significance. *P*‐values <.05 were considered statistically significant.

Ethical approval was granted for our study by the Royal Veterinary College university teaching hospital Ethics and Welfare Committee (URN‐2022‐2104‐03).

## 3 RESULTS

### 3.1 Overview of the study group

A total of 1542 transfusions were administered to 858 dogs during the study period of March 1, 2022 to November 30, 2022. Fourteen centers were involved in the study, 6 from the United Kingdom, 7 from the United States, and 1 from Australia. Institutions comprised 11 academic institutions and 3 private referral centers (all from United Kingdom). Of the 858 dogs that received a transfusion, 363 received more than 1 transfusion (Table 1).

**TABLE 1:** The number of transfusions received by dogs grouped by product type.

No. of TF	pRBC	Whole blood	Plasma products
	No of dogs (%)	No of dogs (%)	No of dogs (%)
1	383 (66.7)	47 (74.6)	199 (60.5)
2	133 (23.2)	10 (15.9)	84 (25.5)
3	27 (4.7)	6 (9.5)	26 (7.9)
4	18 (3.1)	0 (0)	10 (3.0)
5	7 (1.2)	1 (0)	8 (2.4)
6	3 (0.5)	2 (0)	2 (0.6)
7	1 (0.2)	3 (0)	0 (0)
8	2 (0.3)	4 (0)	0 (0)
Total	574	63	329

Abbreviations: No, number; pRBC, packed red blood cells.

From 1542 transfusion events, 108 TRs occurred in 99 dogs. No TRALI, anaphylactic or hypocalcemic reactions were reported.

### 3.2 TR incidence and associations

#### 3.2.1 Packed red blood cells

A total of 878 pRBC units were administered to 574 dogs. More than 1 pRBC unit was received by 191 dogs. The number of pRBCs received by each dog is shown in Table 1.

Indications for pRBCs are listed in Table 2. Blood loss was the most common indication (45%). Data regarding the characteristics of the pRBC units and transfusion events can be seen in Table 3. Leukoreduced units were transfused in 229 out of the 864 (26.5%) cases where data were available. The median volume administered was 12 mL/kg (range, 1‐55.5), and the median age of a unit was 16 days (range, 0‐44).

**TABLE 2:** Listed indications for 1542 blood transfusions in dogs by product type.

Categories	Number	%
pRBC		
Blood loss	392	44.6
Hemolysis	368	41.9
Ineffective erythropoiesis	63	7.2
Extracorporeal treatments	24	2.7
More than 1 indication	5	0.6
Unknown/other	26	3.0
Total	878	…
Whole blood		
Blood loss	58	68.2
Hemolysis	21	24.7
Ineffective erythropoiesis	2	2.4
Unknown/other	4	4.7
Total	85	…
Plasma product		
Colloidal and volume support	220	41.0
Coagulopathy	165	30.7
Other hemorrhage	98	18.2
Extracorporeal treatments	38	7.1
More than 1 indication	11	2.0
Unknown/other	5	0.9
Total	537	…
Platelet products		
ITP	6	46.2
Hemorrhage excluding TP	6	46.2
Extracorporeal treatments	1	3.4
TP of other cause	0	0.0
Total	13	…
Albumin products		
Colloidal and volume support	29	100

Abbreviations: ITP, immune thrombocytopenia; pRBC, packed red blood cells; TP, thrombocytopenia.

**TABLE 3:** Descriptive characteristics for transfusions grouped by product type.

	pRBC			Whole blood			Plasma products		
Categorical variables	Yes	No	Total	Yes	No	Total	Yes	No	Total
Previous transfusion (RBC ≥4 d ago)	159	582	741	23	59	82	n/a	…	…
DEA‐1–matched unit and recipient	644	118	762	63	12	75	191	133	324
Major cross‐match performed	220	563	783	18	62	80	n/a	…	…
Prestorage leukoreduction	229	635	864	4	76	80	173	347	520
General anesthetic during transfusion	107	707	814	13	69	82	62	438	500

Continuous variables	Median (IQR)	Total	Median (IQR)	Total	Median (IQR)		Total
Volume administered (ml/kg)	12 (8.6‐16.2)	870	16.4 (10.3‐20.1)	85	9.8 (7.1‐16.1)		529
Age of unit (d)	16 (9‐25)	795	1 (0‐9.25)	78	n/a	…	…

Administration method	Number	Number	Number
Fluid pump	596	64	363
Syringe driver	170	12	188
Gravity	89	7	16
Pressure bag	3	2	1
Combination	1	0	1

Abbreviations: d, days; DEA, dog erythrocyte antigen; GA, general anesthesia; IQR, interquartile range; n/a, not applicable; pRBC, packed red blood cell; RBC, red blood cell.

**FIGURE 1:**
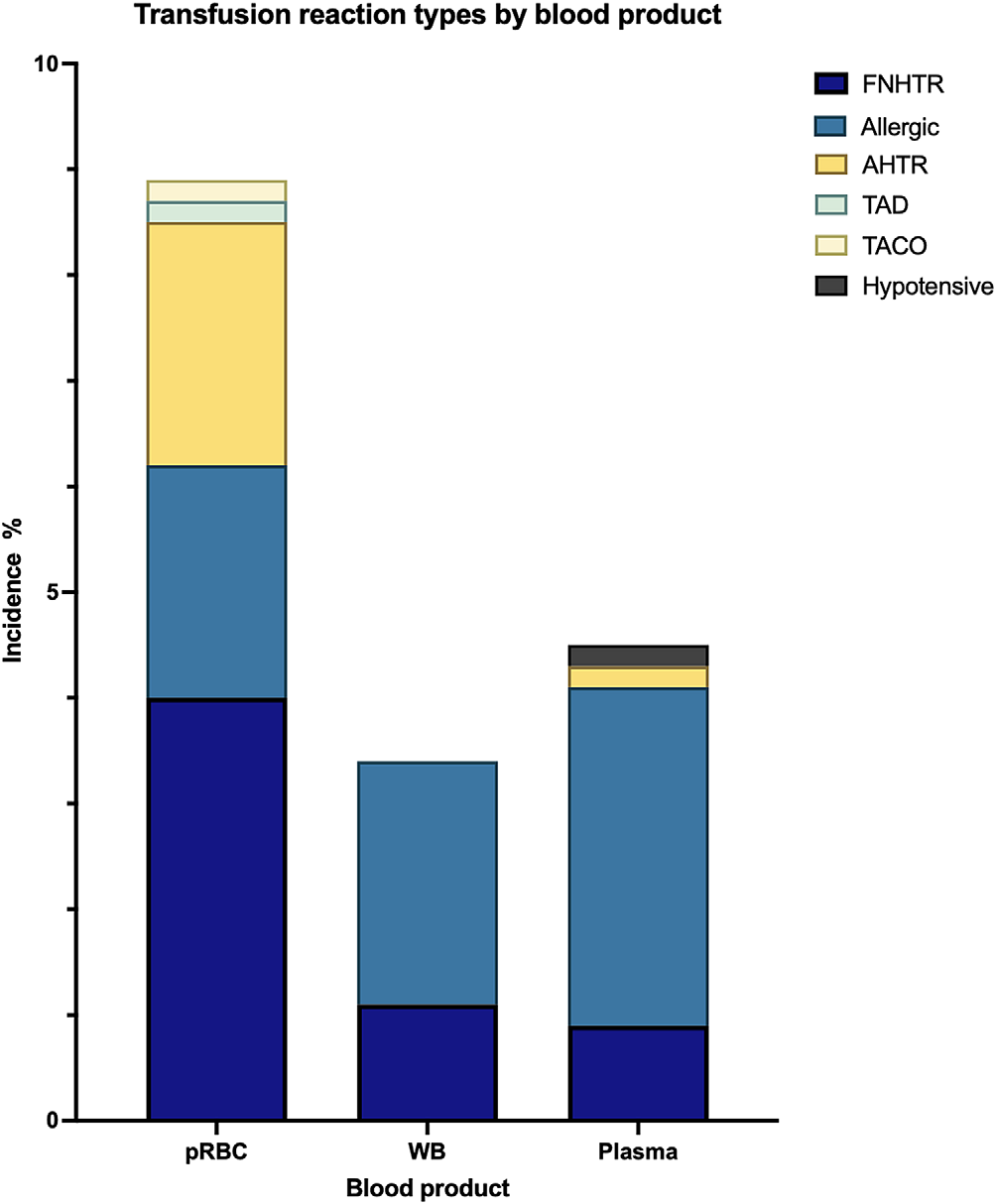
Stacked bar chart showing the relative incidences of each reported category of transfusion reactions for each blood product type in 1542 blood transfusions in dogs. AHTR, acute hemolytic transfusion reaction; FNHTR, febrile nonhemolytic transfusion reaction; pRBC, packed red blood cell; TACO, transfusion‐associated circulatory overload.; TAD, transfusion‐associated dyspnea; WB, whole blood.

TRs occurred in 78/878 pRBC transfusion events, giving an incidence of 8.9% (95% CI 7.0‐11.1). The most common reactions were FNHTR (4% [95% CI 2.8‐5.5]), AHTR (2.3% [95% CI 1.4‐3.5]) and allergic (2.2% [95% CI 1.3‐3.4]) (Figure 1).

The age of the pRBC unit was positively associated with the odds of developing a TR (OR per day 1.06 [95% CI 1.04‐1.09]). Compared with transfusions involving pRBC units of <14 days of age, transfusion of pRBC 21 to 28 days of age had an aOR of 2.25 (95% CI 1.04‐4.90) for any reaction. pRBCs 28 to 44 days of age had adjusted odds of 5.59 (95% CI 2.85‐10.96) for any TR and 4.10 (95% CI 1.58‐10.65) for FNHTR and an OR of 15.2 (95% CI 3.35‐68.70) for AHTR (Table 4).

**TABLE 4:** Association between potential transfusion reaction risk factors and the incidence of all, febrile nonhemolytic, acute hemolytic and allergic transfusion reactions associated with canine packed red blood cell transfusions.

Outcome	Risk factor	OR comparison	No. of TRs/total in group	Univariable	*P* value	Mulitvariable	*P* value
				OR (95% CI)		aOR (95% CI)	
Any TR	Age of unit	14‐21 d v 0‐14 d	13/177 v 17/333	1.47 (0.70‐3.11)	.31	1.37 (0.61‐3.08)	.44
n = 78		21‐28 d v 0‐14 d	16/142 v 17/333	2.36 (1.16‐4.82)	.02*	2.25 (1.04‐4.90)	.04*
		28‐44 d v 0‐14 d	30/143 v 17/333	4.94 (2.62‐9.29)	<.001*	5.59 (2.85‐10.96)	<.001*
		Continuous variable (d)	…	1.06 (1.04‐1.09)	<.001*	…	…
	Leukoreduction	Yes v no	26/229 v 51/635	1.47 (0.89‐2.42)	.13	…	…
	Major cross‐match	Yes v no	23/220 v 42/563	1.45 (0.85‐2.47)	.17	…	…
	DEA‐1 type matched	Yes v no	58/644 v 7/118	1.57 (0.67‐3.53)	.28	…	…
	Transfusion naïvety	RBC ≥4 d previously v not	14/159 v 51/582	1.01 (0.54‐1.87)	.99	…	…
	Type of anemia	Hemolysis v blood loss	41/368 v 32/392	1.41 (0.87‐2.29)	.17	…	…
	GA	Yes v no	2/107 v 67/707	0.18 (0.04‐0.75)	.02*	0.18 (0.04‐0.78)	.022*
	Administration method	Pump v gravity	52/549 v 5/89	1.61 (0.62‐4.14)	.33	…	…
		Syringe driver v gravity	16/160 v 5/89	1.75 (0.62‐4.93)	.29	…	…
	Vol of pRBC administered	Increase in 1 mL/kg administered	…	1.04 (1.01‐1.08)	.008*	1.04 (1.00‐1.08)	.05*
FNHTR	Age of unit	14‐21 d v 0‐14 d	6/177 v 9/333	1.26 (0.44‐3.61)	.66	1.50 (5.09‐4.43)	4.61
n = 35		21‐28 d v 0‐14 d	7/142 v 9/333	1.87 (0.68‐5.12)	.23	2.29 (0.81‐6.51)	.12
		28‐44 d v 0‐14 d	12/143 v 9/333	3.30 (1.36‐8.01)	.02*	4.10 (1.58‐10.65)	.004*
		Continuous variable (d)	…	1.05 (1.01‐1.08)	.006*	…	…
	Leukoreduction	Yes v no	9/229 v 25/635	1.0 (0.46‐2.17)	.99	…	…
	Major cross‐match	Yes v no	14/220 v 19/563	1.95 (0.96‐3.95)	.07*	1.83 (0.87‐3.84)	.112
	DEA‐1 type matched	Yes v no	29/644 v 2/118	2.74 (0.64‐11.62)	.17	…	…
	Transfusion naïvety	RBC ≥4 d previously v not	6/159 v 25/582	0.87 (0.35‐2.17)	.77	…	…
	Type of anemia	Hemolysis v blood loss	17/368 v 15/392	1.22 (0.60‐2.47)	.59	…	…
	GA	Yes v no	0/107 v 33/707	…	.02*	…	…
	Administration method	Pump v gravity	20/549 v 3/89	1.00 (0.29‐3.43)	.99	…	…
		Syringe driver v gravity	7/160 v 9/89	1.21 (0.30‐4779)	.79	…	…
	Vol of pRBC administered	Increase in 1 mL/kg administered	…	1.03 (0.99‐1.08)	.15	…	…
AHTR	Age of unit	14‐21 d v 0‐14 d	1/177 v 2/333	0.94 (0.09‐10.44)	.96	…	…
n = 20		21‐28 d v 0‐14 d	4/142 v 2/333	4.80 (0.87‐26.50)	.07	…	…
		28‐44 d v 0‐14 d	12/143 v 2/333	15.16 (3.35‐68.67)	<.001*	…	…
		Continuous variable (d)	…	1.11 (1.06‐1.16)	<.001*	…	…
	Leukoreduction	Yes v no	8/229 v 12/635	1.88 (0.76‐4.66)	.17	…	…
	Major cross‐match	Yes v no	3/220 v 12/563	0.64 (0.18‐2.7)	.49	…	…
	DEA‐1 type matched	Yes v no	9/644 v 4/118	0.40 (0.12‐1.33)	.14	…	…
	Transfusion naïvety	RBC ≥4d previously v not	3/159 v 12/582	0.91 (2.56‐3.28)	.90	…	…
	Type of anemia	Hemolysis v blood loss	8/368 v 11/392	0.77 (0.331‐1.94)	.58	…	…
	GA	Yes v no	1/107 v 14/707	0.46 (0.06‐3.59)	.46	…	…
	Administration method	Pump v gravity	9/549 v 2/82	0.67 (0.14‐3.14)	.61	…	…
		Syringe driver v gravity	3/160 v 2/82	0.764 (0.12‐4.67)	.77	…	…
	Vol of pRBC administered	Increase in 1 mL/kg administered	…	1.04 (0.99‐1.10)	.13	…	…
Allergic	Age of unit	14‐21 d v 0‐14 d	5/177 v 5/333	1.91 (0.55‐6.68)	.31	…	…
n = 19		21‐28 d v 0‐14 d	5/142 v 5/333	2.39 (0.68‐8.40)	.17	…	…
		28‐44 d v 0‐14 d	4/143 v 5/333	1.89 (0.50‐7.14)	.35	…	…
		Continuous variable (d)	…	1.03 (0.99‐1.08)	.15	…	…
	Leukoreduction	Yes v no	9/229 v 10/635	2.56 (1.03‐6.38)	.04*	2.36 (0.91‐6.13)	.08
	Major cross‐match	Yes v no	4/220 v 9/563	1.14 (0.35‐3.74)	.83	…	…
	DEA‐1 type matched	Yes v No	16/644 v 1/118	2.98 (0.39‐22.70)	.29	…	…
	Transfusion naïvety	RBC ≥4d previously v not	3/159 v 12/582	0.91 (0.256‐3.28)	.89	…	…
	Type of anemia	Hemolysis v blood loss	14/368 v 4/392	3.836 (1.25‐11.76)	.02*	3.42 (1.10‐10.59)	.03*
	GA	Yes v no	0/107 v 17/707	…	.15	…	…
	Administration method	Pump v gravity	16/685 v 0/89	…	.25	…	…
		Syringe driver v gravity	2/170 v 0/89	…	.55	…	…
	Vol of pRBC administered	Increase in 1 mL/kg administered	…	1.06 (1.01‐1.11)	.03*	1.05 (0.99‐1.11)	.120

*Note*: Odds ratios (ORs), aORs, and *P*‐values from single variable and multiple logistic regression.

Abbreviations: AHTR, acute hemolytic transfusion reaction; CI, confidence interval; d, days; DEA, dog erythrocyte antigen; FNHTR, febrile nonhemolytic transfusion reaction; GA, general anesthesia; pRBC, packed red blood cells; TR, transfusion reaction; vol, volume.

* Statistically significant associations.

Leukoreduction of pRBC was not associated with the odds of developing a TR, FNHTR, AHTR, or allergic reaction (Table 4). Transfusion reactions were observed in 26/229 (11.4%) leukoreduced (LR) transfusions versus 51/635 (8.0%) nonleukoreduced (nLR) transfusions.

Multiple other factors were also associated with incidence of TR (Table 4). The odds of developing a TR were higher for every additional 1 mL/kg dose of pRBC administered (aOR 1.04 [95% CI 1.00‐1.08]). Being under GA at any time during a pRBC transfusion was associated with lower odds of any TR (aOR 0.18 [95% CI 0.04‐0.78]). Dogs with hemolytic anemia had higher odds of developing an allergic TR compared with dogs with blood loss (aOR 3.42 [95% CI 1.10‐10.59]; Table 4). Dog erythrocyte antigen (DEA)‐1 type‐matching and performing a cross‐match were not significantly associated with the odds of AHTR (Table 4). In the sensitivity analyses, exclusion of dogs with multiple TRs, and adding center to regression models did not change these results.

#### 3.2.2 Whole blood transfusions

A total of 85 WB transfusions were administered to 63 dogs. Sixteen dogs received more than 1 transfusion (Table 1). The indications for transfusion are shown in Table 2, and data regarding the WB units are in Table 3. Transfusion reactions occurred in 3/85 WB transfusion events giving an incidence of 3.5% (95% CI 0.7‐10.3). These consisted of 2 allergic reactions and 1 FNHTR in 3 dogs (Figure 1).

#### 3.2.3 Plasma products

Five hundred thirty‐seven plasma units were administered to 329 dogs. Fresh‐frozen plasma was the most frequently administered plasma product (449), followed by SFP (42), cryoprecipitate (18), cryo‐supernatant (16), and hyperimmune plasma (12). One hundred thirty dogs received more than 1 unit of plasma product. Table 1 documents the number of transfusions received.

Indications for plasma transfusions are shown in Table 2, with units given for colloidal and volume support being the most frequent (41%). Plasma units' characteristics and transfusion events are summarized in Table 3. Leukoreduction was performed in 173 out of 520 cases where data were available. The median volume administered was 9.8 mL/kg (range, 0.42‐104.3).

TRs occurred in 24/537 plasma transfusion events, giving an incidence of 4.5% (95% CI 2.9‐6.6). Allergic reactions were most frequent at 3.2% (95% CI 1.8‐5.1) with FNHTR at 0.9% (95% CI 0.03‐2.2; Figure 1).

The incidence of TRs did not differ among plasma products. In univariable analysis, being DEA‐1 type matched (OR 0.61 [95% CI 0.15‐2.39]), the volume administered (OR 0.98 [95% CI 0.94‐1.03]), and being under GA (OR 1.13 [95% CI 0.26‐5.07]) were not associated with the odds of a TR.

#### 3.2.4 Platelet products

A total of 13 platelet transfusions were administered to 9 dogs. Six dogs received a single transfusion, 2 received 2 transfusions, and 1 received 3. Refrigerated platelet concentrate was administered in 7 instances, frozen leukoreduced platelet concentrate in 4, and single dogs received lyophilized canine platelets and platelet rich plasma. Indications for platelet administration are shown in Table 2. Two allergic TRs occurred in 2 dogs who both received refrigerated platelet concentrate, giving a TR incidence of 15.4% (95% CI 1.9‐55.6).

#### 3.2.5 Canine albumin products

Twenty‐nine albumin transfusions were administered to 25 dogs. Twenty‐two dogs received a single transfusion, 2 received 2 units, and 1 received 3 units. All transfusions were given for colloidal or volume support. One allergic TR occurred, giving an incidence of 3.4% (95% CI 0.1‐19.2).

### 3.3 Trs—clinical presentations and management

#### 3.3.1 Allergic TR

Of the 41 allergic TRs, 21 had cutaneous signs, 16 had acute and new‐onset vomiting, 1 had both vomiting and cutaneous signs, and 2 had acute and new‐onset diarrhea. Treatment included antihistamines in 22 cases, maropitant in 6, and dexamethasone in 1. In 1 case the transfusion was permanently discontinued with no other drug treatment administered.

#### 3.3.2 Acute hemolytic TRs

Of the 21 AHTRs, 10 were febrile (≥39.2°C, 102.5°F) with an increase of at least 1°C during the transfusion, 6 had hemolysis noted on routine monitoring with no change detected on clinical examination, 3 had cardiovascular destabilization documented during the transfusion, and 1 was febrile with cutaneous signs. One reaction was detected during a plasma transfusion; however, the dog had received 3 pRBC units in the preceding 24 hours. Single dogs received an antihistamine, lidocaine, and maropitant because of reported signs of nausea. Five of the 16 transfusions were permanently stopped, 11 were continued without pause.

#### 3.3.3 Febrile nonhemolytic TRs

Four of the 41 FNHTRs experienced vomiting. The median maximum temperature was 40.1°C (range, 39.2‐41.5°C). Treatment included paracetamol (acetaminophen) in 3 dogs, antihistamines in 2, and maropitant in 1. Active cooling was instigated in 1 case. Three cases had their transfusion permanently stopped.

#### 3.3.4 Hypocalcemia/citrate toxicosis

No cases fulfilling the TRACS definition of hypocalcemia/citrate toxicosis were documented.

#### 3.3.5 Respiratory reactions

Two TACO and 2 TAD reactions were documented; all occurred with pRBC products. The 2 TACO events occurred in the same animal but on separate dates. The 2 episodes of TAD self‐resolved following cessation of the transfusion. The incidence of respiratory TRs to pRBC transfusion was 0.45% (95% CI 0.1%‐1.2%).

#### 3.3.6 Hypotensive reactions

One hypotensive TR was documented with a decrease in both systolic and mean blood pressure of 50 mm Hg within 30 minutes of starting a plasma transfusion. The hypotension did not resolve on cessation of the transfusion. No hemolysis or other systemic cause for the hypotension was identified.

#### 3.3.7 TRs and GA

The 183 dogs that received all or part of their transfusion while anesthetized had a lower incidence of TR of 2.2% (95% CI 0.6%‐5.6%). The reactions documented were 2 allergic, a single AHTR, and a single case of TAD.

### 3.4 Transfusion practices and other complications

#### 3.4.1 Blood product time at room temperature, pauses, and duration of transfusions

Blood products were at room temperature for >4 hours in 19% of the transfusion events. Pauses were documented in 105 (6.8%) transfusions, with 82 (78%) of these subsequently being restarted and transfusion of the unit completed. The most frequent cause was a possible TR check (63/115, 55%), followed by an error of administration (such as cannula dislodgement; 24/115, 21%), drug administration (7/115, 6%), nursing care related (5/115, 4%), to allow for diagnostics (4/115, 4%), and unknown (2/115, 2%). Transfusions were stopped because of death of the recipient in 10 (0.6%) events.

The median time to administer the whole transfusion was 4 hours 54 minutes (4:24‐5:35) including blood products that were split with subunits kept refrigerated until use to avoid prolonged exposure to room temperature.

#### 3.4.2 Premedication

Premedication was given to decrease the likelihood of a TR before 16 transfusions in 14 animals. Calcium gluconate was administered in 8 cases, antihistamine in 6, and corticosteroids in 2. Because of the low number receiving premedication, no further statistical analysis was performed.

#### 3.4.3 Other transfusion‐related complications

New onset hypothermia was documented in 7 cases. Four cases had been under GA for part of their transfusion. The median transfusion dose received in these cases was 20 mL/kg (range, 14‐107) and the median weight of these recipients was 26.2 kg (range, 0.9‐33.4).

Other complications that occurred over the study period included 1 each of administering an expired unit, transfusing the incorrect unit and the observation of a blood clot in the administration set of a pRBC unit.

#### 3.4.4 Death 24 hours after transfusion

Data assessing death 24 hours after transfusion end were available for 1481 transfusion events. One hundred eighty‐six dogs died within 24 hours following 256 transfusion events meaning 21.7% of dogs administered a transfusion in our study died within 24 hours of their last transfusion. One hundred thirty‐eight dogs had received 1 transfusion, and 34, 10, 2, and 2 dogs received 2, 3, 4 and 6 transfusions, respectively. An increase in the volume of pRBC administered was the only factor associated with increased odds of death 24 hours after transfusion (OR 1.06 [95% CI 1.03‐1.10], *P* < 0.001). The occurrence of a TR was not associated with the odds of death in the following 24 hours (OR 1.16 [95% CI 0.68‐1.97], *P* = 0.60).

## 4 DISCUSSION

Our study documents the incidence of acute TRs in a large, multicenter, prospective surveillance study using recently published consensus definitions.^2^ The primary aim of the study was to report the incidence of TRs using well‐defined criteria. Transfusion reaction incidence was 8.9% and 4.5% for pRBC and plasma transfusions, respectively. The most reported TRs were FNHTRs for pRBCs and allergic reactions for plasma.

Fever is the most commonly reported adverse reaction to blood product transfusion in veterinary medicine with FNHTR being the most common cause.^2^, ^3^, ^8^, ^10^, ^11^, ^12^ In our study, FNHTRs were the most frequent TR in pRBCs (4%), with a much lower incidence (0.9%) in plasma units. Febrile nonhemolytic TR incidences of 3% to 24% and 0.7% to 5% have been reported in dogs receiving RBC‐containing products and plasma products, respectively.^8^, ^12^ Results of our study are consistent with the lower rates of FNHTR documented in recent literature.^9^, ^12^ It is interesting to note that human hemovigilance monitoring report lower incidences of FNHTR (0.1%‐0.62%).^1^, ^13^

Consistent with findings in the human literature, allergic TRs were more common in platelet (16.7%) and plasma (3.2%) recipients than RBC‐containing transfusion recipients (2.2%).^13^ However, as the wide CIs in our study indicate, the incidence for platelet transfusions should be cautiously interpreted. Platelet TR incidence varies markedly in the veterinary literature, with reported ranges of 0% to 14%.^14^, ^15^, ^16^, ^17^ Reports of allergic TRs have been nonstandardized, with cutaneous, gastrointestinal, and respiratory signs that meet the TRACS definition reported separately or variably.^2^, ^18^ The incidence of allergic TRs overall (2.7%) and allergic TRs in dogs receiving plasma (3.2%) in our study are comparable to recent larger studies (2.7%‐4.2%).^8^, ^12^ Plasma and platelet products contain higher concentrations of protein allergens such as IgA and haptoglobin, purported to lead to a higher incidence of TRs over pRBC units.^13^

Acute hemolytic transfusion reactions have decreased in frequency in people in the 21st century because of improved compatibility testing, with immunologic AHTRs reported in 1:200 000 transfusion events.^19^, ^20^ Acute hemolytic transfusion reactions might also be because of nonimmunologic mechanisms secondary to mechanical, osmotic, or thermal factors, with these in turn being affected by storage conditions, age, and administration methods. Veterinary AHTR definitions have differed significantly, with temperature increases and markers of hemolysis variably included. Reported incidences vary from 0% to 6.3% in dogs.^2^, ^3^, ^8^, ^9^, ^10^ Our study reports an AHTR incidence in pRBC transfusions of 2.3%; although this is lower than some previous studies, it demonstrates that AHTRs are significantly more common in dogs than people. Dog erythrocyte antigen (DEA)‐1 type‐matching and performing a cross‐match were not significantly associated with decreased odds of AHTR, but the CIs were wide. It is important to note that the definition of a non–DEA‐1 type‐matched transfusion in our study included DEA‐1–positive dogs receiving DEA‐1 negative blood products. Cross‐matching transfusion‐naïve dogs before their 1st transfusion is controversial, with 1 retrospective study showing a greater mean change in hematocrit in dogs who received cross‐matched pRBCs.^21^ It was not possible to consistently evaluate hematocrit rise in our study. The TRACS guidelines “suggest that major cross‐matching might not be necessary for transfusion‐naïve dogs.”^7^ The increase in AHTRs found in older pRBC units might suggest that the AHTRs reported in our study are secondary to nonimmunologic causes.

Acute respiratory TRs have a reported incidence of 1% in people.^1^ Our study in dogs demonstrated a lower incidence of 0.45%, of which all were during pRBC transfusions. The 2 TACO events occurred in the same dog on different dates and responded favorably to furosemide administration. No cases of TRALI were identified; this was not unexpected, given its previously reported rarity in veterinary medicine. It might also represent the difficulty in differentiating TRALI from other causes of respiratory distress in a critically ill population.^2^, ^22^ The incidence of TRALI reported in human studies varies widely, with variable definitions used; a recent 2019 consensus statement aimed to improve detection and hemovigilance.^23^, ^24^ TAD is a novel term in the veterinary literature and was developed in human medicine to classify respiratory TRs that were not classifiable as TACO or TRALI.^2^ As such, the incidence of TAD has not been reported before, and the incidence of 0.2% reported here provides a baseline for future studies. Previous incidences of respiratory reactions range from 2% to 6.3%; the inclusion of the term “acute respiratory distress” in the TRACS definitions, rather than the increased respiratory rate used in other studies, likely accounts for the lower incidences found.^2^, ^3^, ^11^

No cases consistent with the TRACS definition for hypocalcemia/citrate toxicosis were recorded. This lack of identified cases is because of the stringent definition. A probable case requires a massive transfusion, evidence of hepatic dysfunction, and consistent biochemical changes to be present. A “definite” citrate toxicosis must have these factors plus consistent clinical signs.^2^

The incidences of FNHTR, AHTR, and allergic TRs reported in our study are all higher than those reported in people.^1^, ^19^ The reasons for these differences are unclear, but might be because of innate species differences, or because of more advanced prevention strategies being used in people which are not common in veterinary medicine. These include stringent pretransfusion checks and policies, more in‐depth type‐matching, screening for recipient antibodies, solvent detergent treatment of plasma and washing of RBCs.^19^

A secondary aim of our study was to evaluate whether transfusion policies were associated with the odds of developing TRs. The administration of pRBC units stored for over 21 days (compared with units aged 0‐14 days) was associated with a higher odds of a TR (aOR 2.25 [95% CI 1.04‐4.90]), although a significant difference was not observed for individual TRs. However, units aged 28‐44 days were associated with higher odds of overall TR (OR 4.94 [95% CI 2.62‐9.29]), FNHTR (OR 3.30 [95% CI 1.36‐8.01]) and AHTR (OR 15.16 [95% CI 3.35‐68.67]) compared with units aged 0‐14 days. A 2022 study demonstrated an increased risk of TR for every additional day of pRBC storage (OR 1.03), comparable to our study (OR 1.06 [95% CI 1.04‐1.09]).^9^ However, unlike the study by Hann et al., increased odds were not seen in dogs with hemolysis versus other causes of anemia.^11^ A 2018 Cochrane review found no difference in mortality rate for people administered shorter or longer stored RBC products, however, the definition of shorter and longer duration varied in the studies examined.^25^ The data presented in our study suggest that hospital blood banks should carefully manage stock to minimize the aging of units whilst not wasting donated units.^26^

The volume of transfused blood product was associated with the odds of developing a TR, with the administration of each additional 1 mL/kg of pRBC associated with higher odds of any TR of 4% (95% CI 1.00‐1.08) and an allergic TR of 6% (95% CI 1.01‐1.11). Previous veterinary studies have demonstrated increased transfusion‐related complications with an increased RBC dose.^3^, ^10^, ^11^ One study showed increased incidence of hemolytic but not allergic TRs with increasing blood product dose.^10^ There was no association between dose and AHTRs in our study, but statistical power was reduced because of the low AHTR incidence. Our study supports the adoption of a conservative rather than a liberal transfusion strategy. This aligns with human studies that have demonstrated a lack of inferiority or worsened outcomes in specific intensive care unit (ICU) patients randomized to a restrictive transfusion protocol.^27^, ^28^ Increasing the volume of pRBCs administered was also associated with an increased odds of death at 24 hours after transfusion, however this could well reflect large volume resuscitation with blood products that can be required in certain critical conditions, rather than a higher dose being directly associated with increased odds of death.

Studies comparing prestorage LR and nLR RBC products have provided discordant findings on the effect of LR on TR incidence in people and dogs. The administration of LR RBC units was associated with a lower incidence of FNHTRs in some human studies and a recent large retrospective study in dogs.^9^, ^29^, ^30^ However, another randomized controlled trial in humans and other studies in dogs have failed to demonstrate a decreased risk of TR when using LR blood.^18^, ^31^ A Cochrane review of prospective human studies failed to find evidence to support or reject the use of LR pRBC.^32^ Our study did not identify a positive effect of LR on FNHTR or any TR incidences, although the CIs were wide. Further larger studies are required to improve the precision of this finding. It is likely, however, that if there is a difference in TR incidence with LR, it is small.

Dogs under GA for all or part of the transfusion had lower odds of a TR. Bruce et al. similarly documented a lower TR incidence in dogs administered transfusions between 6 hours before and 4 hours after surgery.^8^ No FNHTRs were detected under GA, suggesting that the GA‐induced hypothermia might mask the incidence, or impede the detection, of these (and potentially other) TRs.

Regarding transfusion practices, 19% of transfusions involved a blood product being at room temperature for >4 hours. The 4‐hour guideline is advised to reduce bacterial proliferation in blood products, although some studies suggest that the effect of this could be minimal.^33^, ^34^ No septic TRs were noted in our study. The fairly high percentage (21.7%) of dogs that died within 24 hours of receiving their last transfusion suggests that a significant portion of dogs in our study were critically ill. It is unknown how many recipients survived to discharge and beyond, making it hard to compare this survival rate with other studies.

Strengths of our study include the collection of a large, prospective, multicenter, multinational dataset alongside the use of new evidence‐based consensus definitions with imputability statements. Transfusion reactions were classified and then agreed upon between 2 authors to improve accuracy, with follow‐up information gathered to ensure the TR was classified as accurately as possible. The exclusion of “possible” TRs increases the ability of our study to identify true TR incidences. However, it might be that some true TRs were excluded from the final data analysis. The documentation of TR incidences with 95% CIs further strengthens the utility of our study when guiding future comparable research.

Our study is not without limitations. Being a large multicenter trial might introduce confounding by institutions with potential variations in blood donor pools, collection methods, blood‐banking protocols, LR filters, cross‐matching methods, administration equipment, transfusion monitoring procedures, TR reporting, and documentation. Not all institutions adopted the AVHTM transfusion monitoring sheet, meaning some data were missing. Various authors logged the information using a standardized proforma, and although final TR classifications were decided according to TRACS guidelines, some variation in data entry might have occurred. Some animals received simultaneous or consecutive transfusions, leading to difficulty in assessing which product resulted in a TR. Although data were prospectively gathered, information regarding cross‐matching, blood typing, and death was still unavailable in some cases. It was not possible to verify whether TRACS algorithm recommended investigations, for instance, for hemolysis, were performed in some cases. This might have led to TRs being missed, misclassified or being assigned a lower imputability grade. Although all transfusions were logged wherever data were available, some products administered in each institution might have been missed. Despite the 1542 transfusions documented, the low incidence of TRs means that our study is underpowered to detect small differences in risk associated with individual exposures. In addition, the use of immunosuppressants in some dogs (eg, those with immune‐mediated disease) might have influenced the ability of animals to generate observable TRs. The small number of platelet and canine albumin transfusions likely reduced the precision of estimated population TR incidences. Finally, data regarding TR severity were not assessed.

## 5 CONCLUSION

Assessed prospectively using TRACS definitions, the incidence of acute TRs in dogs associated with pRBC and plasma transfusions were 8.9% and 4.5%, respectively. Increased pRBC dose and age of products were associated with increased odds of developing a TR. Leukoreduction of pRBCs was not shown to reduce acute TRs. Although these results are observational, they add to evidence that pRBC dose and age are potentially modifiable risk factors for TRs. This information can be used to better inform treatment decisions, communicate risks and benefits associated with transfusions to owners and serve as a benchmark for future research studies.

## CONFLICT OF INTEREST DECLARATION

Authors declare no conflict of interest.

## OFF‐LABEL ANTIMICROBIAL DECLARATION

Authors declare no off‐label use of antimicrobials.

## INSTITUTIONAL ANIMAL CARE AND USE COMMITTEE (IACUC) OR OTHER APPROVAL DECLARATION

Approved by the Royal Veterinary College university teaching hospital Ethics and Welfare Committee (reference number URN 2022 2104‐03).

## HUMAN ETHICS APPROVAL DECLARATION

Authors declare human ethics approval was not needed for this study.

## Supporting information


**Data S1:** Supporting Information.
