# Supplementary material for: A prospective multicenter observational study assessing incidence and risk factors for acute blood transfusion reactions in dogs
Source: J Vet Intern Med. 2024 Sep 6;38(5):2495–506. doi: 10.1111/jvim.17175 (PMC11423481; doi:10.1111/jvim.17175)
Supplement: Supplementary file 1 — Data S1: Supporting Information. [file JVIM-38-2495-s001.pdf]

Transfusion Monitoring Survey

Page 1: Blood product monitoring survey

This site allows you to upload information about a veterinary blood product transfusion. The aim is to use the data submitted to produce a publication to increase veterinary knowledge about transfusion monitoring and transfusion reactions.

Page 2: Page 1

1. What is the name of the institution where this transfusion was administered?

- Auburn
- DWR
- Georgia
- Langford
- Louisiana State University
- MS State
- NDSR
- Queensland
- The Ralph
- RVC
- RDVS
- Tufts
- UW Madison
- Washington state
- Willows

2. What species was the recipient?

- Dog
- Cat

2.a. Was the blood product administered DEA 1 type matched?

- Yes
- No
- Unsure

2.b. Was the blood product administered A/B/AB type matched?

- ○ Yes
- ○ No
- ○ Unsure

3. What is the unique case identifier (case number) of the recipient?

4. What is the patient's weight in kg?

5. What blood product was adminstered?

- ○ PRBC
- ○ FFP
- ○ Whole blood
- ○ Non-FFP Whole plasma
- ● Other

5.a. If you selected Other, please specify:

5.b. Has the patient had a previous red cell containing transfusion more than 2 days ago if a cat or 4 days ago if a dog?

- Yes
- No
- Unsure

5.b.i. Was a major crossmatch performed prior to administration?

- Yes
- No

5.b.i.a. Was the crossmatch compatible?

- [ ] Yes
- [ ] No
- [ ] Unsure

5.b.i.a.i. Add more details here about the incompatible crossmatch if available (severity of incompatabilty etc).

6. What was the date of blood product collection?

Dates need to be in the format 'DD/MM/YYYY', for example 27/03/1980.

(dd/mm/yyyy)

7. Was the blood product leukoreduced prior to storage?

- Yes
- No
- Unsure

8. Was a drug administered prior to the transfusion to decrease the risk of trasnfusion reaction (pre-treatment)?

- Yes
- No

8.a. What drug was administered?

- [ ] Acetaminophen
- [ ] Anti-histamine
- [ ] Corticosteroid
- [ ] Other

8.a.i. What drug was administered?

9. Please provide the date and time of the start of the blood product transfusion

Please enter a date and time in the format 'DD/MM/YYYY HH:MM', for example 27/03/1980 15:43.

(dd/mm/yyyy hh:mm)

10. Which of the following methods of adminstration was used?

- ◯ Fluid pump
- ◯ Gravity
- ◯ Syringe driver
- ◯ Other

10.a. Please specify method of administration

11. Was the patient under general anaesthesia at any point during the transfusion?

- ☐ Yes
- ☐ No

Page 3: Page 2

12. Were any of the following noted during the period of transfusion administration or in the 24 hours after the end of the transfusion? Tick all that apply.

- ☐ Acute fever over 39C (102.5F) AND 1C (1.8F) above temperature at transfusion starting point
- ☐ Acute respiratory distress
- ☐ Acute tachycardia and/or hypotension
- ☐ Angioedema/urticaria/pruritis
- ☐ Change in mentation
- ☐ Hypocalcaemia
- ☐ Hypothermia
- ☐ Vomiting (acute and new onset)
- ☐ Diarrhoea (acute and new onset)
- ☐ Other possible transfusion reaction
- ☐ None of the above

12.a. Was plasma haemolysed at the time of tachycardia/hypotension?

- ○ Yes
- ○ No
- ○ Not checked

12.a.i. Was the patient

- ☐ Receiving a massive transfusion? (1 whole blood volume in 24hours, 90ml/kg dogs 60ml/kg cat)
- ☐ Having on-going severe haemorrhage?
- ☐ Showing signs of anaphylaxis? (Tachycardia, hypotension, GI signs and/or gall bladder wall oedema)
- ☐ Showing acute respiratory distress?

12.a.ii. Please give further information about: a. The severity of tachycardia/hypotension b. When the tachycardia/hypotension was noted c. Any further investigation performed not detailed previously d. Any treatment administered

12.b. Were new bilateral pulmonary infiltrates present on CT, radiography or ultrasound?

- Yes
- No
- Unsure
- Not checked for

12.b.i. Was there evidence of plasma haemolysis at the time of respiratory distress?

- Yes
- No
- Not checked for

12.b.ii. Was there evidence of cardiac overload on echocardiography or NT-proBNP measurement?

- Yes
- No
- Not checked
- Other

12.b.ii.a. If you selected Other, please specify:

12.b.ii.b. Please give details of echo measurements or NT-proBNP value if taken.

12.b.ii.c. Please give further information about: a. The respiratory distress b. When the respiratory distress was noted c. Any further investigation performed not detailed previously d. Any treatment administered

12.c. Was there plasma haemolysis at the time of the fever?

- Yes
- No
- Not checked
- Unsure

12.c.i. Was acute respiratory distress noted?

- Yes
- No

12.c.i.a. Did the patient have acute cardiovascular deterioration at the time of the fever?

- ◯ Yes
- ◯ No

12.c.i.a.i. Please give details of severity and parameters

12.c.i.a.ii. Please give further information about: a. The peak fever b. When the fever was noted c. Any further investigation performed not detailed previously d. Any treatment administered

12.c.ii. Was there evidence of

- ☐ Incorrect blood type
- ☐ Cross match incompatibility
- ☐ Out of date unit
- ☐ Inappropriate transfusion administration set up
- ☐ Other concern with unit or administration method
- ☐ None of the above

12.c.ii.a. Please give further details about suspected incompatability or inappropriate administration method.

12.c.ii.a.i. Please give further information about: a. The peak fever b. When the fever was noted c. Any further investigation performed not detailed previously d. Any treatment administered

12.d. Please give further information about: a. The clinical signs seen b. When the clinical signs were seen c. Any further investigation performed d. Any treatment administered.

13. Was the transfusion paused at any time?

- Yes
- No

13.a. Please give details about why the transfusion was paused and how long the transfusion was stopped for.

14. Was the whole planned transfusion volume administered?

- Yes

○ No

14.a. Please give details as to why planned volume was not administered.

15. What volume of blood product was administered (ml)?

Page 4: Page 3

16. Please provide the date and time of the end of the transfusion

Please enter a date and time in the format 'DD/MM/YYYY HH:MM', for example 27/03/1980 15:43.

(dd/mm/yyyy hh:mm)

17. Did any of the following occur during the transfusion?

- ☐ Blood product at room temperature for greater than 4 hours
- ☐ Intravenous catheter displacement
- ☐ Incorrect blood product rate administration
- ☐ Other transfusion associated complication
- ☐ None of the above

17.a. Please give more details about the complication including its timing and any actions taken.

18. What was the underlying disease process meaning blood product transfusion was required?

- ○ Anticoagulant rodenticide induced coagulopathy
- ○ Bone marrow disease
- ○ IMHA / PIMA

- IMTP
- Surgical blood loss
- Trauma induced blood loss
- Other
- Unknown

18.a. Please give more details about the underlying disease process

19. Was the patient alive at 24 hours post transfusion

- Yes
- No

20. Did the patient have another blood product transfusion in the 24 hours prior to or 24 hours after this blood product transfusion

- [ ] Yes
- [ ] No

20.a. Have you registered the other transfusion(s) or are you planning to do so immediately after this entry?

- [ ] Yes
- [ ] No

21. Please add any other relevant information here.

Page 5: Final page

Thank you so much for submitting this case to the blood product transfusion registry.
